# Supplementary material for: Controversy of Peptide Cyclization from Tripeptide
Source: Molecules. 2021 Jan 13;26(2):389. doi: 10.3390/molecules26020389 (PMC7828492; doi:10.3390/molecules26020389)
Supplement: Supplementary file 1 [file molecules-26-00389-s001.pdf]

molecules

Supplementary information

Controversy of Peptide Cyclization from Tripeptide

Chung-Yin Lin [1,2,*], Subrata Chakraborty [3], Chia-Wei Wong [3] and Dar-Fu Tai [4,*]

[1] Medical Imaging Research Center, Institute for Radiological Research, Chang Gung University/Chang Gung Memorial Hospital, Taoyuan 333423, Taiwan
[2] Department of Nephrology and Clinical Poison Center, Chang Gung Memorial Hospital, Taoyuan 333423, Taiwan
[3] Department of Chemistry, National Dong Hwa University, Hualien 974003, Taiwan; cpapu2000@yahoo.com (S.C.); linny5782@hotmail.com (C.-W.W.)
[4] Department of Life Science, National Dong Hwa University, Hualien 974003, Taiwan
* Correspondence: winwood5782@gmail.com (C.-Y.L.); dftai@gms.ndhu.edu.tw (D.-F.T.); Tel.: +886-3-211-8800 (ext.3865) (C.-Y.L.)

Citation: Lin, C.-Y.; Chakraborty, S.; Wong, C.-W.; Tai, D.-F. Controversy of Peptide Cyclization from Tripeptide. *Molecules* **2021**, *26*, 389. https://doi.org/10.3390/molecules26020389

Academic Editor: Theodore Tselios
Received: 16 November 2020
Accepted: 5 January 2021
Published: 13 January 2021

Publisher's Note: MDPI stays neutral with regard to jurisdictional claims in published maps and institutional affiliations.

Copyright: © 2021 by the authors. Licensee MDPI, Basel, Switzerland. This article is an open access article distributed under the terms and conditions of the Creative Commons Attribution (CC BY) license (http://creativecommons.org/licenses/by/4.0/).

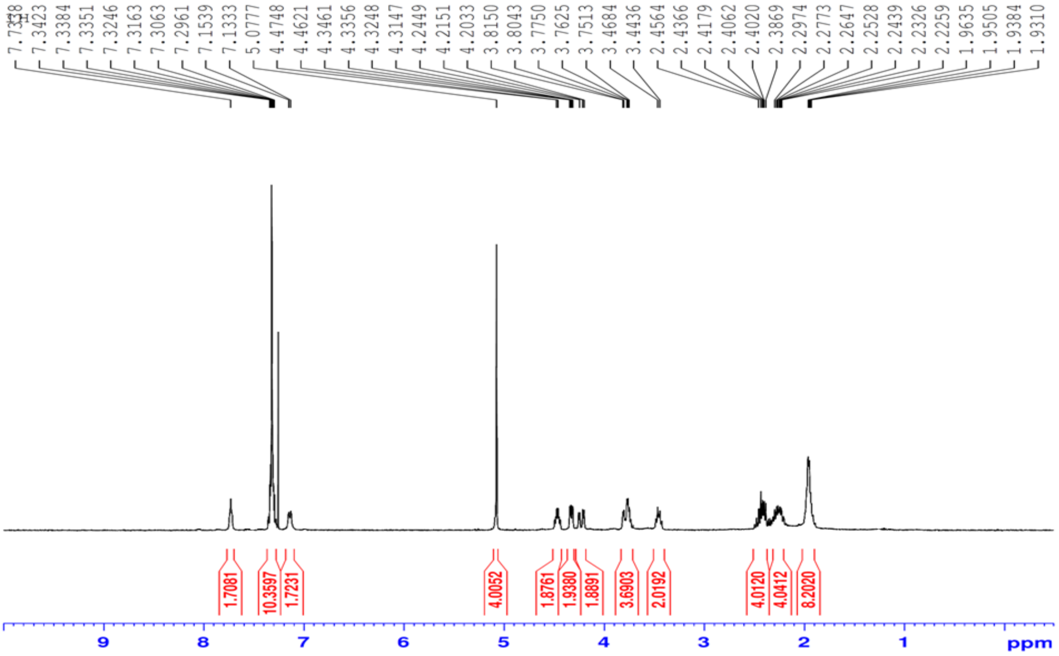

Figure S1. ^{1}H NMR spectrum of *cyclo*(Gly-L-Pro-L-Glu(OBn))$_2$ **7** in $CDCl_3$.

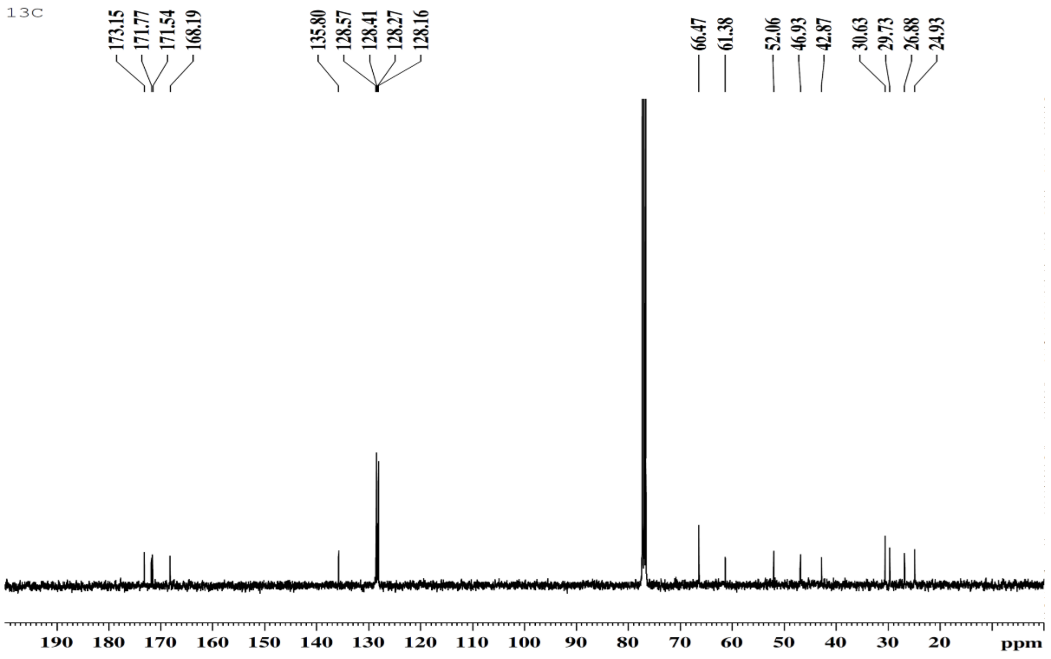

Figure S2. ^{13}C NMR spectrum of *cyclo*(Gly-L-Pro-L-Glu(OBn))$_2$ **7** in $CDCl_3$.

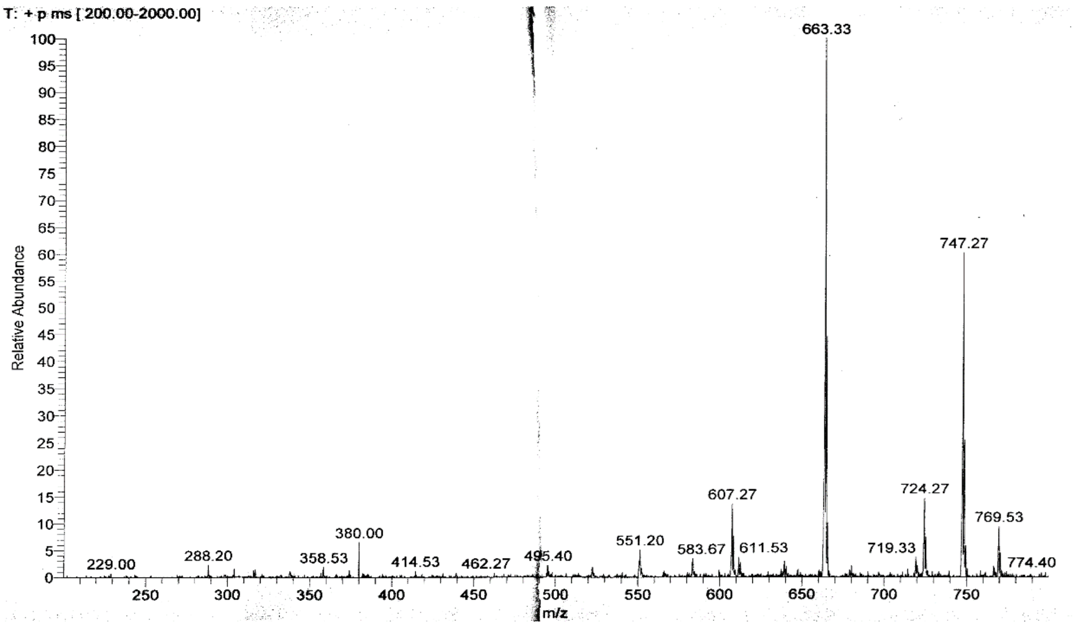

Figure S3. HRMS(ESI) spectrum of *cyclo*(Gly-L-Pro-L-Glu(OBn))2**7.**

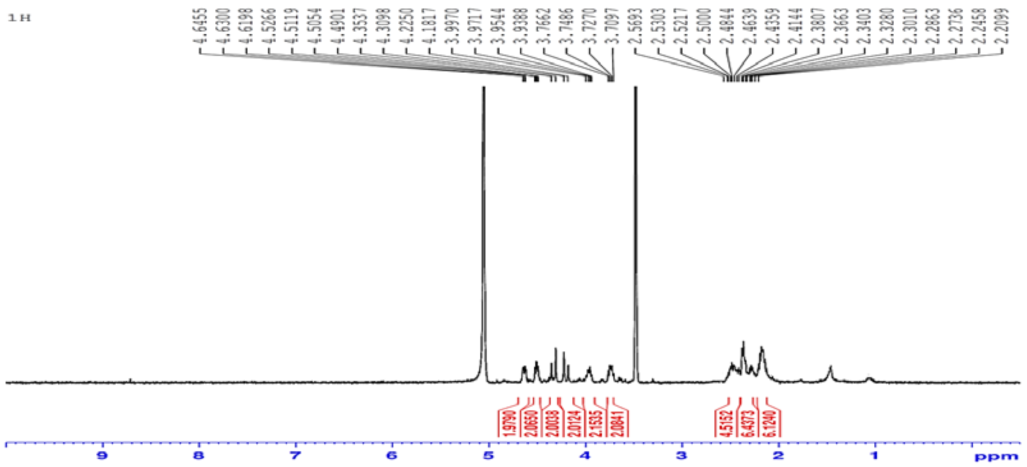

Figure S4. ^{1}H NMR spectrum of *cyclo*(Gly-L-Pro-L-Glu)2 **2** in CD_3OD/D_2O.

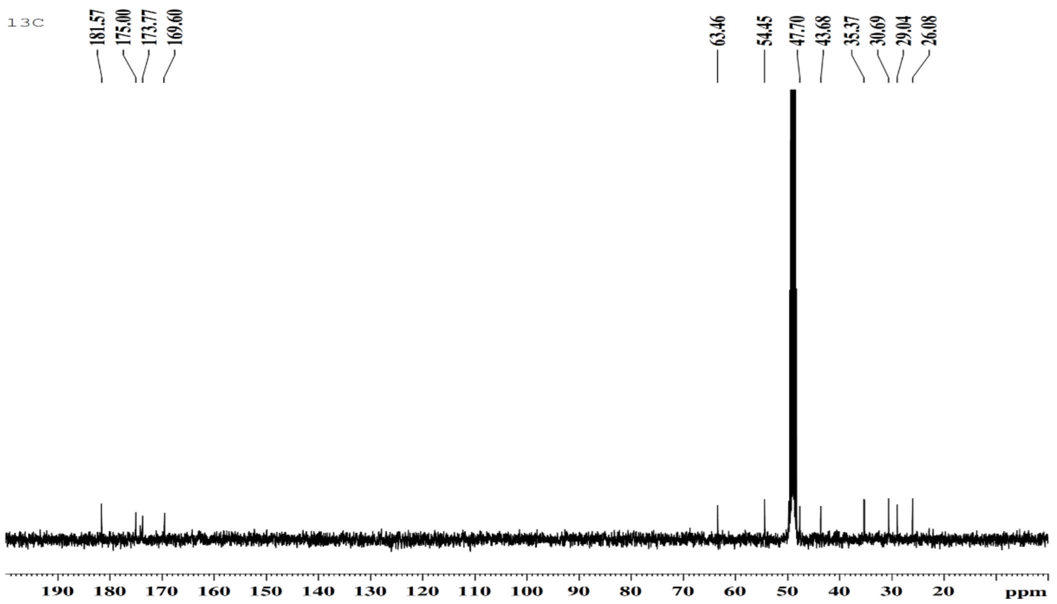

Figure S5. ^{13}C NMR spectrum of *cyclo*(Gly-L-Pro-L-Glu)$_2$ **2** in CD_3OD/D_2O.

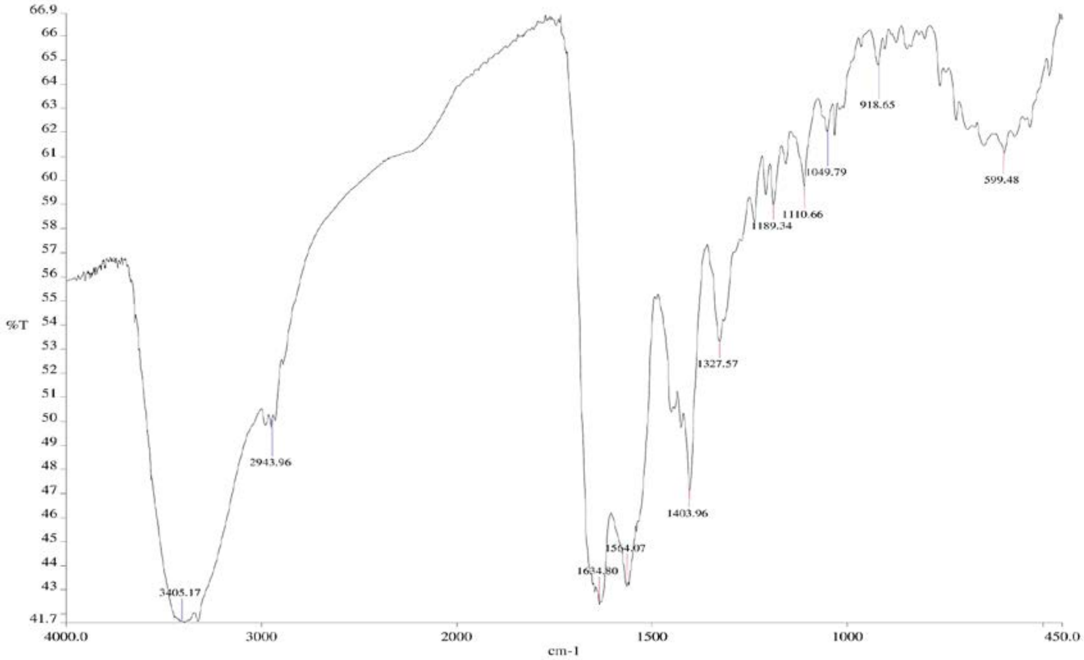

Figure S6. IR spectrum of *cyclo*(Gly-L-Pro-L-Glu)$_2$ **2.**

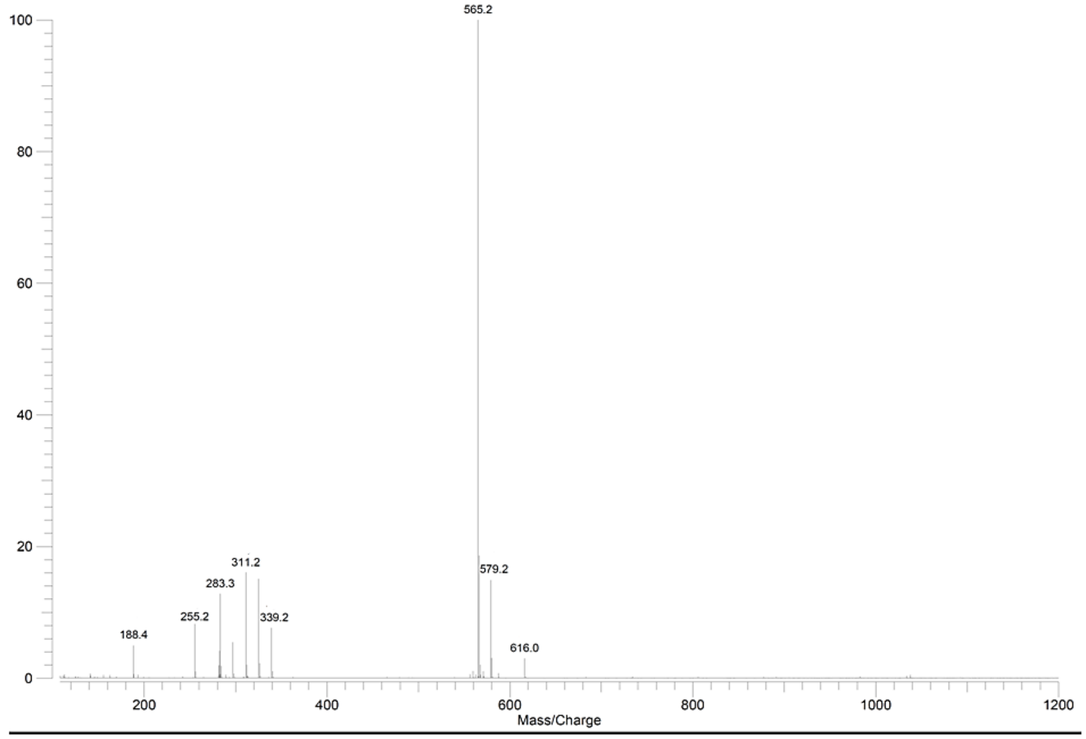

Figure S7. HRMS(ESI) spectrum of *cyclo*(Gly-L-Pro-L-Glu)2**2.**

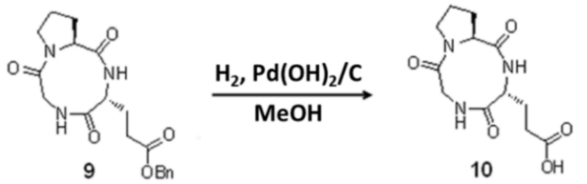

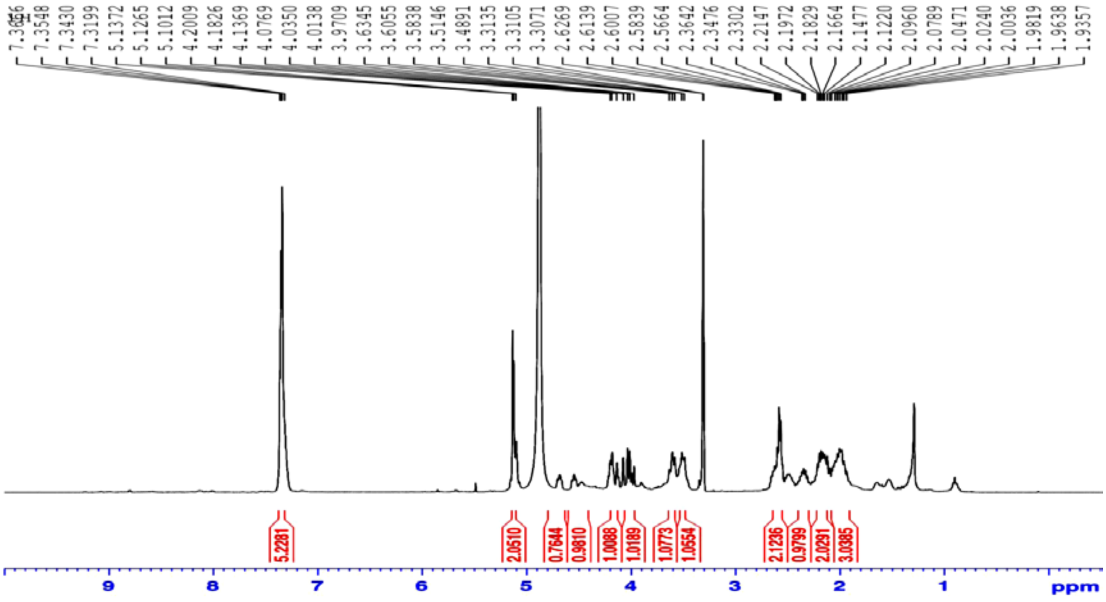

Figure S8. ^{1}H NMR spectrum of *cyclo*(Gly-L-Pro-D-Glu(Bn)) **9** in $CDCl_3$.

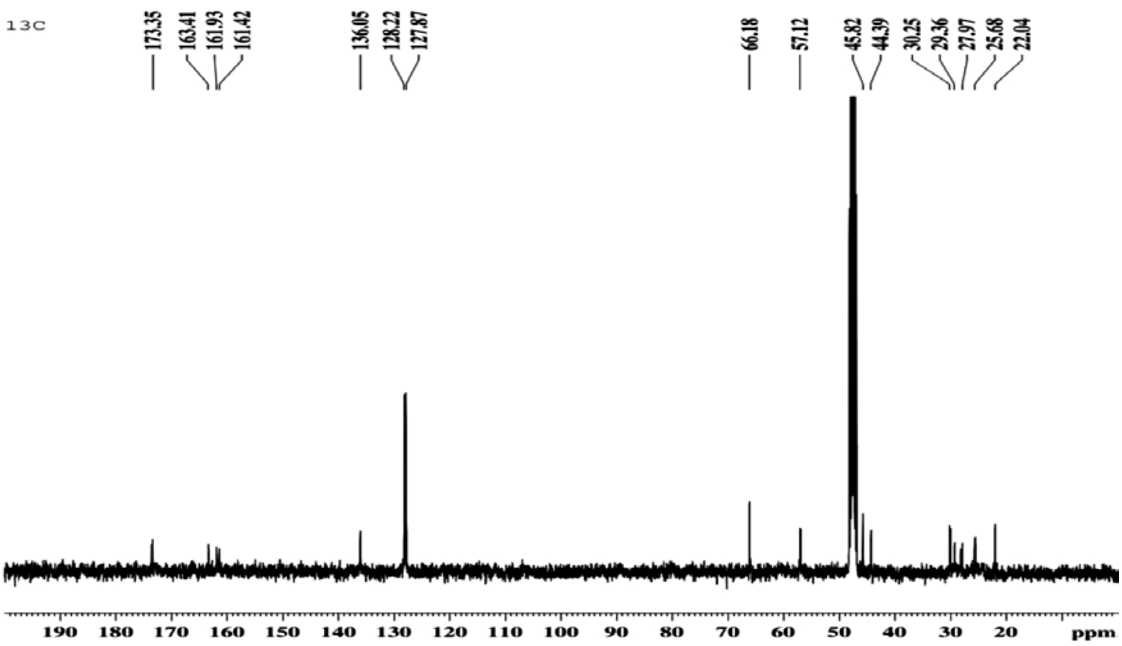

Figure S9. ^{13}C NMR spectrum of *cyclo*(Gly-L-Pro-D-Glu(Bn)) **9** in $CDCl_3$.

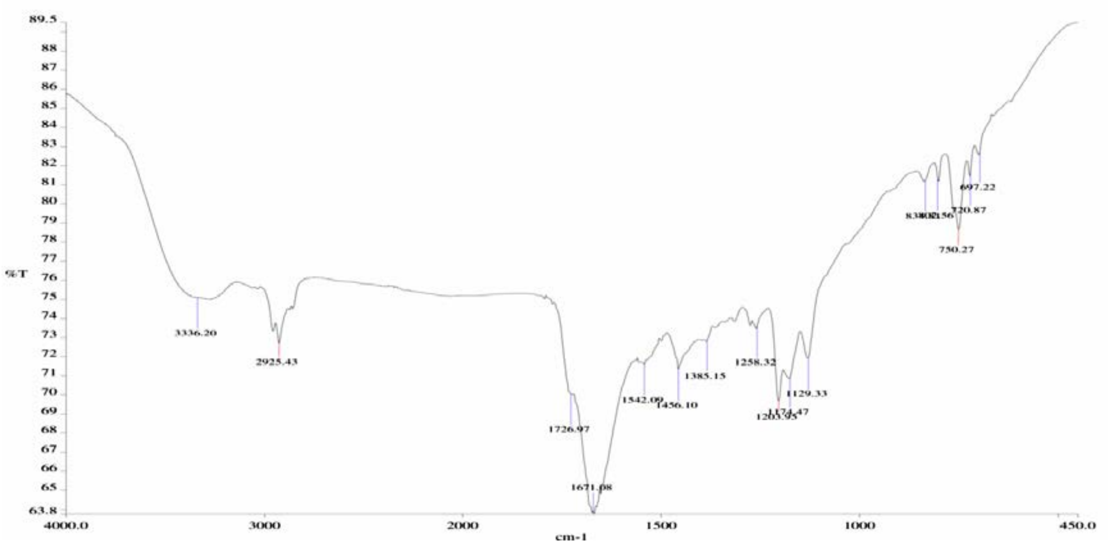

Figure S10. IR spectrum of *cyclo*(Gly-L-Pro-D-Glu(Bn)) **9**.

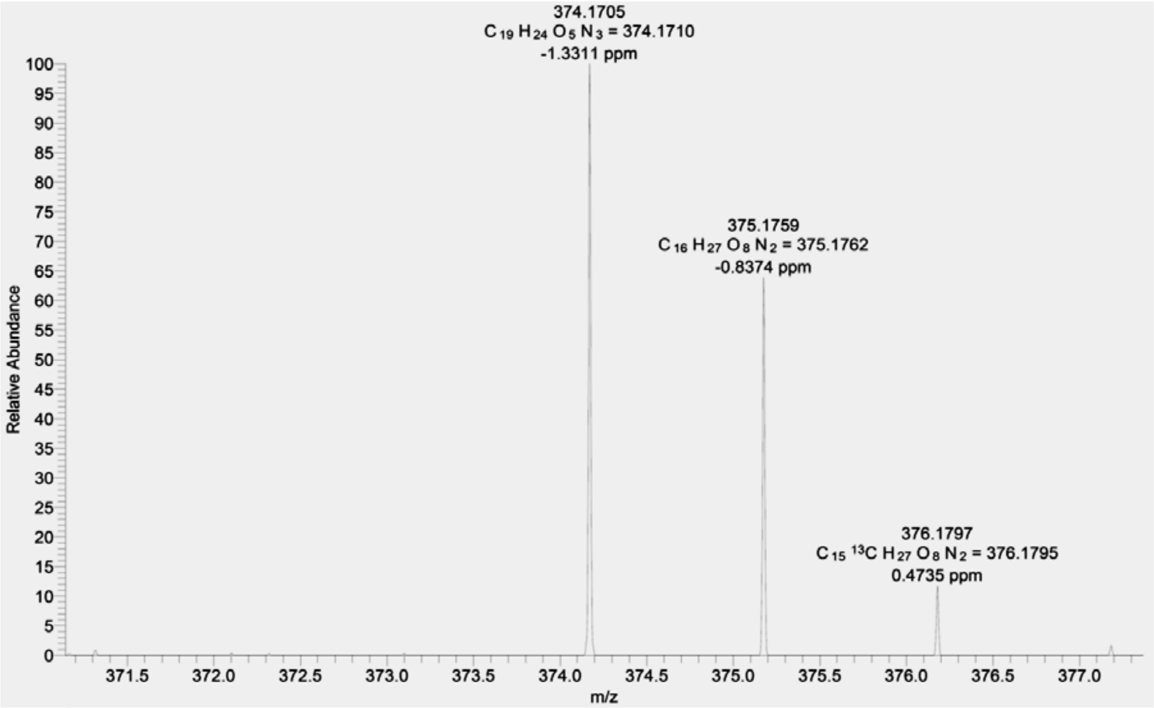

Figure S11. HRMS(ESI) spectrum of *cyclo*(Gly-L-Pro-D-Glu(Bn)) **9**.

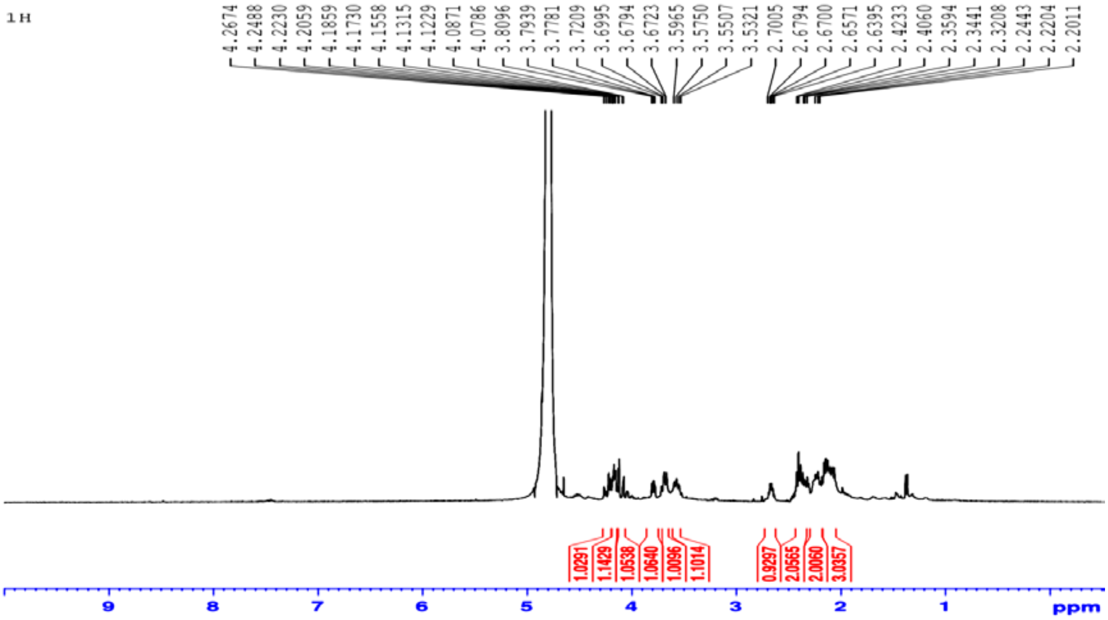

Figure S12. ^{1}H NMR spectrum of *cyclo*(Gly-L-Pro-D-Glu) **10** in D_2O.

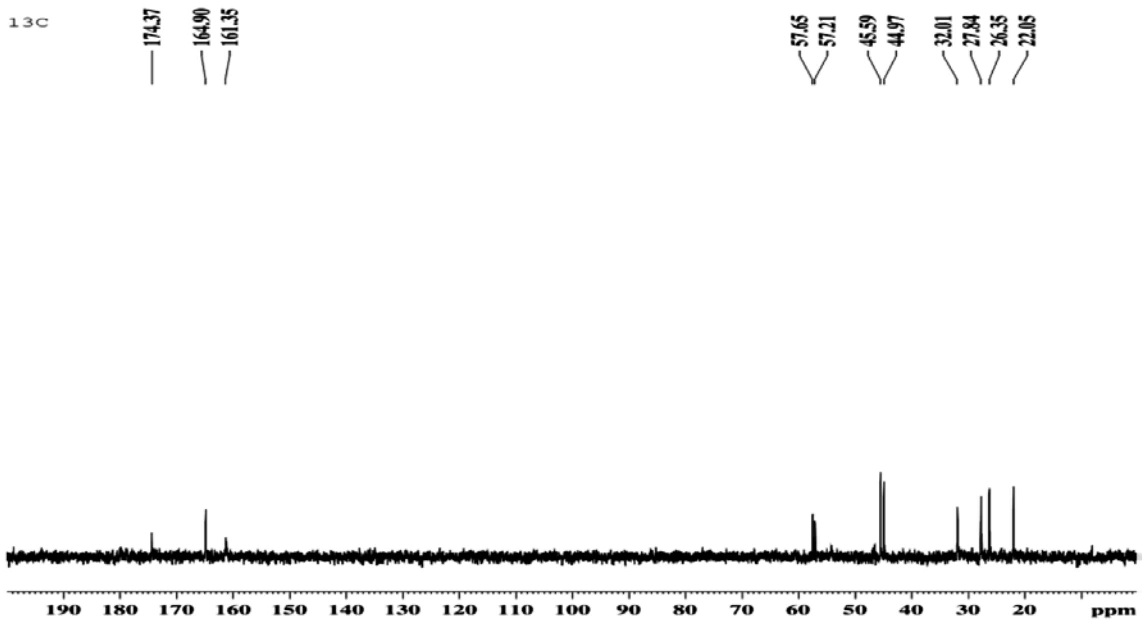

Figure S13. ^{13}C NMR spectrum of *cyclo*(Gly-L-Pro-D-Glu) **10** in D_2O.

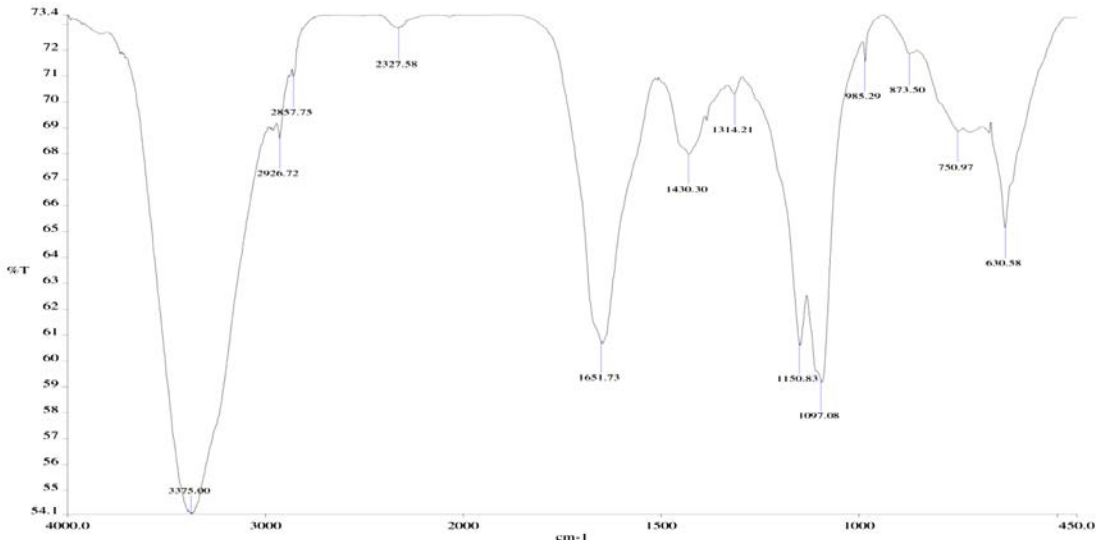

Figure S14. IR spectrum of *cyclo*(Gly-L-Pro-D-Glu) **10**.

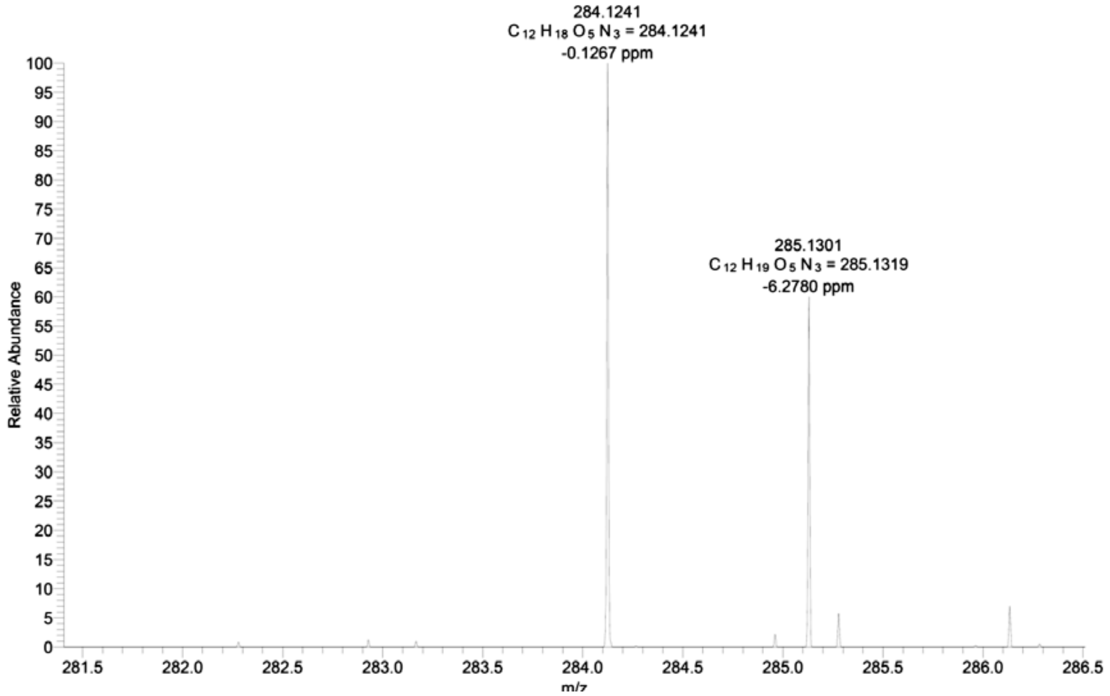

Figure S15. HRMS(ESI) spectrum of *cyclo*(Gly-L-Pro-D-Glu) **10**.
